# Supplementary material for: Usability and acceptability of a two-way texting intervention for post-operative follow-up for voluntary medical male circumcision in Zimbabwe
Source: PLoS One. 2020 Jun 16;15(6):e0233234. doi: 10.1371/journal.pone.0233234 (PMC7297350; doi:10.1371/journal.pone.0233234)
Supplement: S1 Data — (DOCX) [file pone.0233234.s001.docx]

**Reducing provider workload while preserving patient safety: a 2-way texting intervention in Zimbabwe’s voluntary medical male circumcision program**

Version 1, March 2017

**Principal Investigator: Prof Mufuta Tshimanga**

**Collaborating Organizations:**

Zimbabwe Community Health Intervention Research Project: ZICHIRe (Zimbabwe)

I-TECH, University of Washington (USA)

UZ-UCSF Collaborative Research Programme

Ministry of Health and Child Care (Zimbabwe)

Cooperative Agreement title: Scaling up Voluntary Male Circumcision to Prevent Transmission in Zimbabwe. Cooperative Agreement #: 5U2GGH000972. Project Period: 5/15/2013 – 3/31/2019

**Study Sponsor:**

National Institutes of Health, USA, under proposal: “PAR-16-292: Reducing provider workload while preserving patient safety: a 2-way texting intervention in Zimbabwe’s voluntary medical male circumcision program”

# I. Investigator Affiliations, Roles and Responsibilities

1. Professor Mufuta Tshimanga, MD, MPH

Affiliation: Department of Community Medicine, University of Zimbabwe, College of Medicine

Role: Principal Investigator

Responsibility: Dr Tshimanga carries the overall end-responsibility of the project, including oversight for the protocol development, implementation, and assurance for the timely reporting and dissemination of study results.

2. Vernon Murenje, MBChB, MSc, I-TECH, Zimbabwe

Affiliation: I-TECH, Zimbabwe.

Role: Co-Principle Investigator

Responsibility: Dr Murenje will oversee and manage all aspects of local implementation and participate in the reporting of results.

3. Caryl Feldacker, PhD, MPH I-TECH, University of Washington

Affiliation: I-TECH, University of Washington.

Role: Principle Investigator

Responsibility: Dr Feldacker carries the end-responsibility of the project from the I-TECH, University of Washington side, including the development and implementation of the study protocol, data collection and analysis, and reporting of results.

4. Professor Scott Barnhart, MD

Affiliation: I-TECH, University of Washington.

Role: Co-Investigator

Responsibility: Prof. Barnhart will help lead the development and implementation of the study protocol and contribute to reporting of results.

5. Mr. Sinokuthemba Xaba, RGN, BSc, MSc

Affiliation: Zimbabwe Ministry of Health and Child Care

Role: Co-Investigator

Responsibility: Mr. Xaba is the National VMMC Coordinator in the HIV and TB Programme of the Zimbabwe Ministry of Health and Child Care. He will be involved with the implementation of the study and will function as the Ministry’s liaison with participating health facilities.

6. Batsirai Makunike-Chikwinya

Affiliation:    I-TECH, Zimbabwe.

Role:           Co-Investigator. Responsibility:

Batsirai Makunike-Chikwinya is the Country Director for I-TECH Zimbabwe. She will also review all study documents and will be involved in the writing and reporting of results.

The Investigators and co-investigators report no conflict of interest or competing interest in the submission, implementation, dissemination or publication of this work.

# 2. STUDY BACKGROUND, PURPOSE AND SUMMARY

Male circumcision (MC) reduces the risk of female-to-male HIV-1 transmission by up to 60% [1-3]. From 2008-2015, 10.4 million voluntary medical male circumcision (VMMC) procedures were performed [4], falling far short of the 20 million needed to reach the UNAIDS/ World Health Organization (WHO) target of 80% coverage in 14 priority countries by 2016 [5]. Reaching the target could avert 3.4 million HIV infections and save $16.5 billion in HIV-related care through 2025 [6]. Although surgical VMMC has been streamlined and made efficient through operational optimization [7], current VMMC follow-up requires at least one in-person visit within 14 days of MC [8-10]. VMMC program guidelines often require multiple, additional visits over the 42-day healing period. **Severe healthcare worker shortages** [11] **combined with rapidly expanding VMMC programs threaten already overburdened healthcare systems by recruiting workers away from facilities or diverting their efforts from other competing patient care needs**.

In pilot studies and controlled trials in sub-Saharan Africa, surgical adverse event (AE) rates from combined moderate and severe AEs range from 0.5% to 8% with few severe AEs resulting in permanent impairment [1-3, 12-19]. Although AE reporting in field settings is challenging [20-22], AE rates averaged 0.8% (range: 0.4%-4.2%) in all southern African countries from 2010-2012 [23]. These low AE rates correspond to 99% of men healing without incident, leading to millions of healthcare visits conducted without cause and needlessly increasing the workload. For men, these unnecessary visits likely create barriers to care such as transportation costs, wait times, and inconvenience [24-29], potentially reducing VMMC uptake. **Empowering men to seek in-person follow-up care only when needed or desired would decrease the burden for both healthcare workers and patients, thereby improving efficiency and reducing both direct and indirect costs for this critical component of combination HIV prevention.**

The spread of mobile telephones throughout sub-Saharan Africa affords an important opportunity to address these inefficiencies and retain quality care. Text messages show immense promise to positively influence health behavior and improve clinical care [30-32]. Most short message service (SMS)-based health promotion efforts use one-way ‘push’ messages, blasting pre-defined messages to many people simultaneously [33-35]. Among VMMC programs, a randomized control trial determined that automated SMS modestly increased adherence to 7-day follow-up visits from 59.7% to 65.4% (RR 1.09, 95% CI 1.00–1.20; p = 0.04) but did not influence resumption of sex during the 42-day healing period (relative risk=1.13, 95% CI 0.91-1.38, p=0.3) [29, 36]. An important weakness of these one-way, blast approaches is the one-size-fits-all approach, removing patients’ ability to communicate back with healthcare workers [37], potentially reducing patient engagement in care.

Two-way texting (2wT) between providers and patients shows promise to provide quality interactive care. In Kenya, patients enrolled in a SMS study significantly increased adherence and viral load suppression, but high-volume, manual texting was time-consuming and not sustainable for healthcare workers [38]. Another Kenyan study engaged patients in important health-related conversations and provided critical health information [39]; however, this intervention tasked the health workers to respond to complicated, open-ended questions, investing considerable time. In 2008 members of this study team pioneered an alternative approach to provide high-quality, efficient, coordinated service by enabling patients or community health workers to exchange ad hoc messages with facility-based nurses in Malawi [40, 41]. **Use of interactive, mobile technology that improves both care quality and care efficiency for providers and patients is warranted.**

Zimbabwe is primed for this type of mobile intervention. In 2014, Zimbabwe reached about 25% of its 1.3 million VMMC target for 2018 [42]. Although some AE underreporting is possible [42], Zimbabwe’s VMMC program is safe with the national VMMC program’s AE rate of 0.35% [42]. Under the current VMMC Zimbabwe Ministry of Health and Child Care (MoHCC) standards, **1 million additional VMMCs in Zimbabwe will translate into** **more than 2** **million unnecessary follow-up visits.** As each VMMC client likely passes through reception, observation, and examination, **the healthcare workload and time lost for VMMC clients healing without complication is likely several times higher.** Moreover, VMMC staff are MoHCC employees who perform most VMMCs in outreach sites (tertiary clinics, schools, communities). Unnecessary follow-up in outreach sites results in additional days away from routine clinic duties, stretching human resources required for myriad other health needs. While healthcare is strained, mobile technology is advancing rapidly: in 2014 approximately 81% of Zimbabweans had a cell phone [43]. This setting with high, unnecessary in-person VMMC follow-up; healthcare worker shortages; and high cell coverage is ideal to test and evaluate a better, low-cost approach to minimizing the burden on both the healthcare system and VMMC clients.


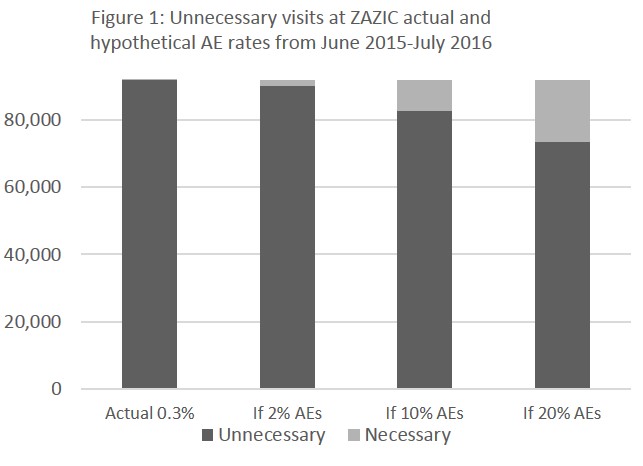
Building upon these previous mHealth initiatives, we seek to implement a two-way texting (**2wT**) intervention that **combines automated and interactive text messaging to maintain safety while dramatically reducing the burden that in-person VMMC follow-up places on weak health systems.** Automated messaging during Days 1-14 after circumcision could track men who are healing without complication, enabling a conservative estimate of more than 80% of clients to avoid unnecessary in-person follow-up at post-operative Days 2, 7 and 42. When a patient responds to an automated prompt message in a way that flags a potential AE, interactive messaging with a nurse would encourage them to promptly seek in-person, follow-up care. 2wT would act as active but efficient surveillance, improving care. By combining automated messaging to screen out cases with no complications and interactive texting to identify potential AEs, this intervention could make the care pathway far more efficient, reducing the costs of VMMC care over time for both providers and patients. **Reduced in-person follow-up could also free these same healthcare workers to perform additional VMMC surgeries.**

As a national VMMC implementing partner, the ZAZIC consortium, led by the University of Washington’s International Training and Education Center for Health (I-TECH) and local implementing partners, The University of Zimbabwe(UZ)-University of California San Francisco Collaborative Research Program, Zimbabwe Association of Church-related Hospitals (ZACH), and Zimbabwe Community Health Intervention Research Project (ZiCHIRe), works in partnership with the Zimbabwe MoHCC to implement VMMC services in 10 districts. ZAZIC’s program is highly productive and safe: between July 2015-June, 2016, ZAZIC conducted 47,905 MCs and 45,989 follow-up visits to find 151 AEs, an AE rate of 0.3%[44]. Adherence to follow-up visits, another indicator of program quality [45], was good. Over 95% of men attended both Day 2 and Day 7 follow-up; more than 30% also had a Day 42 visit to verify complete healing [46]. With such high follow-up visits, **even if 20% of men were reviewed on Days 2 and 7 for high sensitivity of AE detection, almost 74,000 in-person visits were unnecessary and could potentially be replaced with 2wT interactions between patients and providers** (Figure 1). ZAZIC continues to rapidly scale up. In ZAZIC’s largest urban district, Chitungwiza, we have reached only 14% of the intended 80% target for 2018 [44], indicating ZAZIC projected exponential increase over the coming 2 years. Greater efficiency would enable more VMMCs.

# 3. GOAL AND OBJECTIVES

**We propose to maintain patient safety while reducing the substantial VMMC follow-up workload burden** by using two-way mobile phone texts to provide in-person review only for men who indicate a desire or need for follow-up. This tailored approach to post-procedure VMMC care could reduce unnecessary visits and remove barriers for VMMC clients without deterioration of quality care. ZAZIC’s diverse, expert team of researchers from the University of Washington, Seattle, USA; the University of Zimbabwe; Medic Mobile, Nairobi, Kenya; and Zimbabwean partner organizations will test this mHealth intervention. We believe that bi-directional, interactive, text-based short message service (SMS) during the most critical 14 days after circumcision will help men identify and act on any sign of an adverse event, thereby seeking in-person follow-up only when an AE is suspected and reducing unnecessary visits. **Reduced in-person follow-up could also free healthcare workers to perform additional VMMC surgeries.** Using a prospective, randomized control trial (RCT), our intervention compares two groups of clients with cell phones: 1) standard care (control group) and 2) clients who receive and respond to a daily text with in-person follow-up only if desired or if an AE is suspected (intervention). Both arms complete a study-specific, Day 14, in-person, follow-up review for verification of self-reports (intervention) and comparison (control). Our specific objectives are to:

**Objective 1: Determine if 2-way texting can safely reduce VMMC follow-up visits**

Approach: Un-blinded, prospective, non-inferiority, RCT in high-volume facilities providing VMMC. Two-way texting (**2wT**) will provide interactive, text-based follow-up. 2wT men healing without complication could decline in-person follow-up; those with suspected AEs will be referred to in-person care. We will compare the safety outcome of combined moderate or severe AE rate ≤ Day 14 post-VMMC and the workload outcome of average number of in-person follow-up visits between control and intervention arms.

**Objective 2: Estimate the cost savings associated with 2wT over routine VMMC follow-up**

Approach: We will determine the programmatic costs of 2wT from a systems perspective, including the technology [47], healthcare worker costs, and client perspective (travel, text costs, missed work). We will estimate the incremental intervention costs relative to standard practice to quantify gains in healthcare efficiency for scale-up and adoption.

**Objective 3: Assess the acceptability and feasibility of 2wT for further scale-up**

Approach: Qualitative interviews with VMMC healthcare workers and brief quantitative interviews with 2wT clients inform intervention acceptance. Meetings with local researchers and collaborators will further assess feasibility, adaptation, open-source collaborative development, and system integration for replication and sustainability in Zimbabwe and the region.

The proposed mHealth study will establish new, low-cost, technology-supported VMMC follow-up that will streamline in-person care, therefore reducing provider workload and patient burden while maintaining the quality of care.

# 4. Study Design

Study overview**:** Following usability testing with both healthcare workers and 2wT VMMC clients, we will test if two-way texting (2wT) reduces unnecessary follow-up visits without compromising patient safety (Objective 1) using a randomized control trial in large, urban VMMC clinics. We will assess the costs of 2wT from technology, healthcare worker, and client perspectives to determine the costs incurred by MoHCC if adopted for scale-up (Objective 2). We will assess acceptability and feasibility working towards system integration and sustainability in Zimbabwe and beyond (Objective 3).


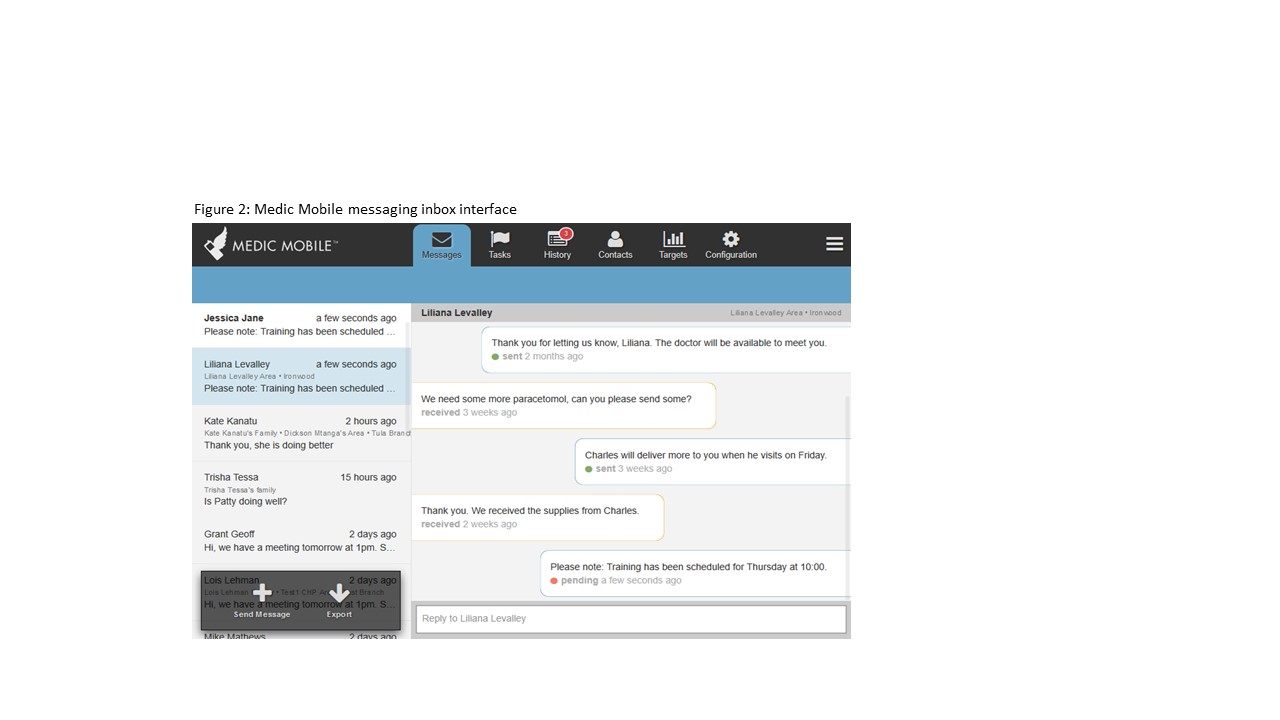
**Objective 1: Technology overview:** Studies that demonstrate the impacts of mHealth interventions too often have evaluated technologies that are made ‘from scratch’ and as a result are not robust enough to merit widespread replication [40, 48]. By partnering with a well-established non-profit mHealth organization, Medic Mobile (<http://medicmobile.org/>), and integrating with their existing software platform, open-source community and current efforts to integrate with existing health information systems (HIS) throughout sub-Saharan Africa, our team and proposed intervention are well positioned to scale up and sustain any promising results. Since 2008, Medic Mobile has been a leader in the global mHealth community [49-51], equipping more than 13,500 health workers serving over 8 million people across 23 countries. The Mobile Medic Toolkit is an Android-based application that supports texting in any language and works with or without internet connectivity on basic phones, smartphones, tablets, and computers [52]. **The existing, well-proven, app-based Toolkit that will be the basis for 2wT provides an automated and prioritized list of upcoming tasks, guiding health worker through actions (e.g., response waiting, referral)**. The Toolkit provides real-time progress indicators such as texting delivery rates and response rates. Data from mobile users are replicated to Medic Mobile web app and analytics tools for real-time response. The platform is highly configurable, currently supporting evidence-backed workflows and program implementation related to ensuring safe deliveries [53], tracking tuberculosis patients [54], boosting immunization rates [55] and monitoring stocks of essential medicines [56]. These adaptable tools are free, open-source, and developed using human-centered design with input from people delivering care in the hardest-to-reach communities. While this technology has never been used in a VMMC program and requires adaptation of the clinical content, the existing 2wT software toolkit already contains the robust messaging features discussed in this proposal (Figure 2). The software adaptation process to alter and test alternative clinical content and the local specifications requires little additional software development. The adaptation process will be completed by collaborator, Holeman, with additional technical and training support provided by Nairobi-based technical experts.

## Recruitment Sites:

Study sites and population: In Zimbabwe, 81% of people already had mobile phone subscriptions in 2014 [57], indicating that the technology infrastructure exists and uptake is high. 2wT will be implemented in existing VMMC sites in the Chitungwiza District, a district purposefully selected for its high VMMC volume clinic locations. Chitungwiza District, a suburb of Harare, has an estimated 300,000 eligible, HIV-negative men ages 15-29; only 14% of eligible men have been circumcised to date. We will implement 2wT in up to 5 ZAZIC VMMC sites in Chitungwiza- Seke North clinic, Seke South clinic, Zengeza clinic, Chitungwiza central hospital and CitiMed hospital, sites averaging 50-200 VMMC/month. As of March, 2017, all VMMC clients are surgical, rather than PrePex device-based, clients.

## Recruitment: Procedure

The City Health Director who manage the national VMMC program at the Provincial level in conjunction with our implementing partners will facilitate access to the clinics/hospitals where ZAZIC provides ongoing support for VMMC. We will also seek permission from the facility manager to conduct the study at that particular clinic.

It would be expected that demand creation would happen in anticipation of the study implementation.

Each site will establish local recruitment and screening methods that operationalize protocol-specified requirements for eligibility determination in a manner that is tailored to and most efficient for the local study setting and target study population. In brief, information about the study will be disseminated through the Zimbabwe Ministry of Health and Child Care (MoHCC), ZAZIC, and HCW at the selected sites. VMMC demand creation as part of routine ZAZIC program practice will support study recruitment. HCW recruited for participation in the study will be reached at their workplaces (the health care facilities) following communication between the study team and the site leadership. VMMC clients will be recruited in the VMMC clinic area. Recruitment will be managed by a specifically-trained research coordinator who receive payment for their roles as part of routine or study work. This research coordinator will meet with VMMC patients to sensitize them about the opportunity to participate in a study of text-based follow-up; those meeting eligibility criteria will be individually informed by 2wT staff about the opportunity to participate in the study. Interested patients will be referred to the site study coordinator for study enrollment and informed consent. This person will meet with patients in a private setting, further explain the study, confirm study eligibility, and seek informed consent. Participants who refuse to enroll in the study will be asked to provide informed consent for data collection about reasons for non-participation.

This activity is being conducted with cooperation from the Ministry of Health and Child Care and implementing partners. This activity will also seek ethical approval from the University of Washington Internal Review Board, Seattle, Washington, USA.

## Eligibility Criteria:

**For VMMC clients (Objective 1):**

Inclusion criteria for VMMC clients are:

1) 18 years or older;

2) possession of own phone at enrollment;

3) provides contact details (phone, physical location);

4) receives surgical VMMC;

5) willing to follow MoHCC VMMC protocols;

6) willing to come in Day 14;

7) Able and willing to respond to a questionnaire administered by phone 42 days after circumcision;

8) No intraoperative adverse event during routine VMMC.

Exclusion criteria:

- Not willing to participate
- Men without cell phones and
- Those who chose PrePex will be excluded as PrePex requires a device-removal visit 7 days after placement and has distinctly different follow-up protocols.

**For health care workers (Objective 3):**

Inclusion criteria are:

Within the 5 study sites, health care workers (HCW) will be eligible to participate in the individual interviews if they:

a) are employees posted at the site;

b) are at least 18 years of age or over;

c) provide health care services to patients as part of the VMMC programs; and

d) are able to provide written informed consent.

Exclusion Criteria:

- Not willing to participate
- Not willing to be recorded

## Study Procedures:

Participant overview:

- 50 men for the pilot. Not randomized. Enrolled into texting. Not included in outcome analysis.
- 722 men in the full study randomized in a 1:1 ratio of texting and control for 361 men in texting and 361 in control (routine care).
- Same recruitment, consent, and follow-up procedures in pilot and intervention as outlined, per group, below

Recruitment and Informed Consent: Each site will establish local recruitment and screening methods that operationalize protocol-specified requirements for eligibility determination in a manner that is tailored to and most efficient for the local study setting and target study population. In brief, information about the study will be disseminated through the Zimbabwe Ministry of Health and Child Care (MoHCC), ZAZIC, and HCW at the selected sites. VMMC demand creation as part of routine ZAZIC program practice will support study recruitment. HCW recruited for participation in the study will be reached at their workplaces (the health care facilities) following communication between the study team and the site leadership. VMMC clients will be recruited in the VMMC clinic area. Recruitment will be managed by a specifically-trained research coordinator. This research coordinator will meet with VMMC patients to sensitize them about the opportunity to participate in a study of text-based follow-up; those meeting eligibility criteria will be individually informed by 2wT staff about the opportunity to participate in the study. Interested patients will be referred to the site study coordinator for study enrollment and informed consent. This person will meet with patients in a private setting, further explain the study, confirm study eligibility, and seek informed consent. Participants who refuse to enroll in the study will be asked to provide informed consent for data collection about reasons for non-participation.

Study preparation: We will conduct a rapid situation analysis with healthcare workers, VMMC clients, and stakeholders to assess suitable responses to VMMC client texts, setting standards for text responses and in-person follow-up. We will modify existing usability surveys for this public health context. A small pilot with 50 VMMC clients who will be enrolled in the texting intervention will include usability testing with both 2wT clients and nurses implementing the 2wT system, illuminating system experiences from both perspectives. Usability results will inform in-box modification, message format preferences (SMS or WhatsApp) and optimal message delivery (timing, frequency, language preferences). Experience from study coordinator and other team members will add detail to inform 2wT adaptation and modification of Standard Operating Procedures before implementation. We will examine local infrastructure (e.g. electricity and cell network), and explore 2wT cost reduction options (e.g., text bundling, free-call back numbers) and adapt accordingly

**Standard** VMMC care (Control arm): For the 361 men randomized into the control arm, ZAZIC follows all MoHCC protocols based on WHO guidelines [8] including routine surgical VMMC follow-up on post-surgery days 2, 7 and 42 (Table 1). Patients may seek care outside scheduled visits for suspicion of AEs at any healthcare facility at any time but most often return to their VMMC site. Referral cards for VMMC clients provide local numbers for patients to text, call, or request a call back for emergencies. A standardized approach is used to assess, identify, and record the severity of AEs [45]. All VMMC care, from assessment of all AEs through complete healing, is provided free to clients from MoHCC. Clients who do not return to the clinic for follow-up on Day 2 or Day 7 are traced: 3 attempts by phone and then up to 3 attempts at in-person tracking after which they are considered lost to follow-up (LTFU) [46]. There is no tracing for Day 42 visits. For the purposes of this study, control arm VMMC clients will be asked to come in on Day 14 for an additional follow-up visit. No active follow-up is provided at Day 14.

VMMC care procedures (2wT arm): We will conduct a prospective, un-blinded, randomized control trial (RCT) among VMMC clients in a 1:1 ratio of control to intervention. Study participants and clinic staff are not masked to treatment. For the 361 men randomized into the intervention arm, men in the 2wT will receive routine VMMC surgical care and counseling, including referral cards for emergencies. 2wT clients will receive automated daily texts from days 1-14 (Table 1 and Figure 3). It is free to receive call and texts; it costs $0.05 to send a SMS in Zimbabwe [58]. If they respond that they suspect no adverse event, no immediate follow-up action will be taken. If a 2wT VMMC client responds affirmatively to any daily text that he suspects an AE, a VMMC nurse will exchange modifiable, scripted texts with the client to determine the symptoms, frequency, and severity. Then, if deemed necessary, the client will be asked to return to clinic the following day or earlier if an emergency is suspected. AE management will adhere to MoHCC standard care. If 2wT patients do not respond to texts on Day 2 or Day 7, the same MoHCC tracing process will be activated, after which they will be considered LTFU. All study participants will be asked to come to the clinic for study-specific, Day 14 follow-up to review healing and verify adverse event reporting. Day 14 was chosen for verification because 95% of all AEs within the ZAZIC VMMC program are reported Day 14 or earlier [44], suggesting that most AEs have occurred by this time point. In a previous field study of AEs, the most common AEs of bleeding and infection were found a mean of 6.7 and 9.0 days, respectively, after VMMC [20], further supporting the 14 day period used in this and a previous study[13]. The Day 14 review will be conducted by routine VMMC providers according to MoHCC review guidelines. At Day 42, we will implement a brief text-based survey with 2wT clients to ascertain complete healing, providing stronger inferences at study completion.


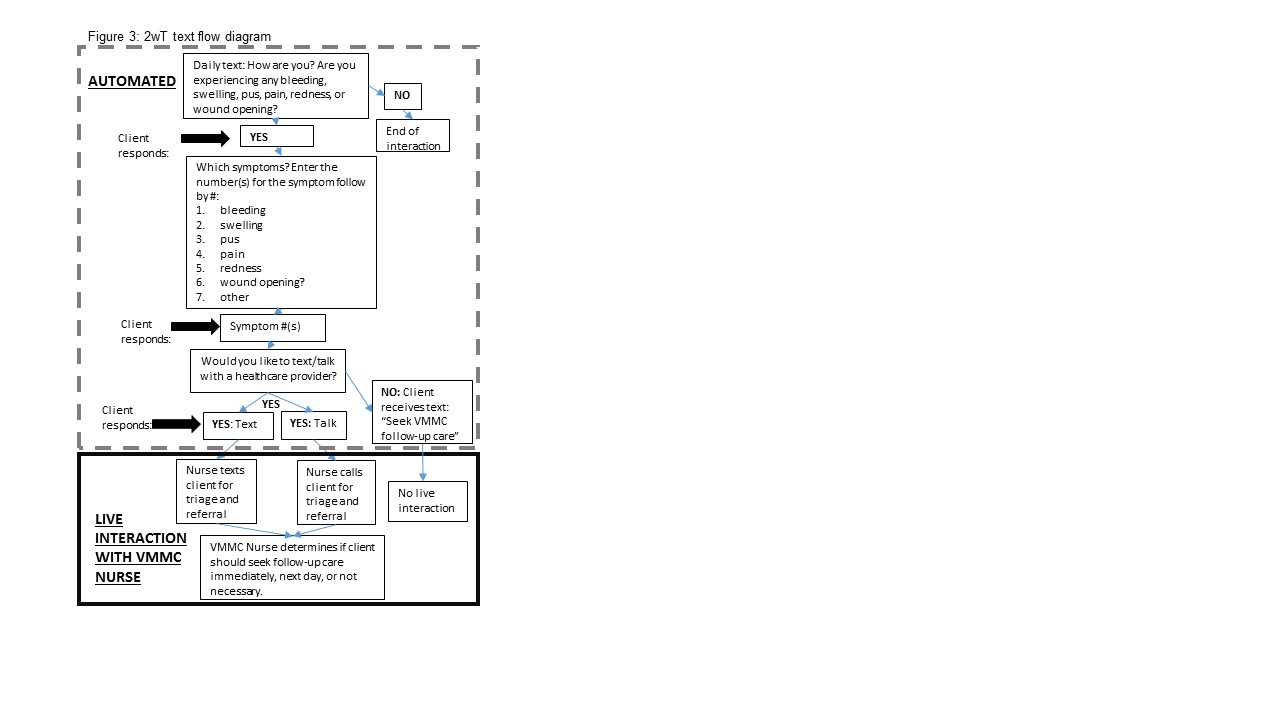
 Participant eligibility and recruitment: VMMC client recruitment, voluntary consent, and enrollment will be managed by the on-site 2wT study coordinator before VMMC surgery. Eligibility criteria for VMMC clients are: 1) 18 years or older; 2) possession of own phone at enrollment; 3) provides contact details (phone, address, next of kin); 4) requests surgical VMMC; 5) willing to follow MoHCC VMMC protocols; 6) willing to come in Day 14; 7) willing to respond to a
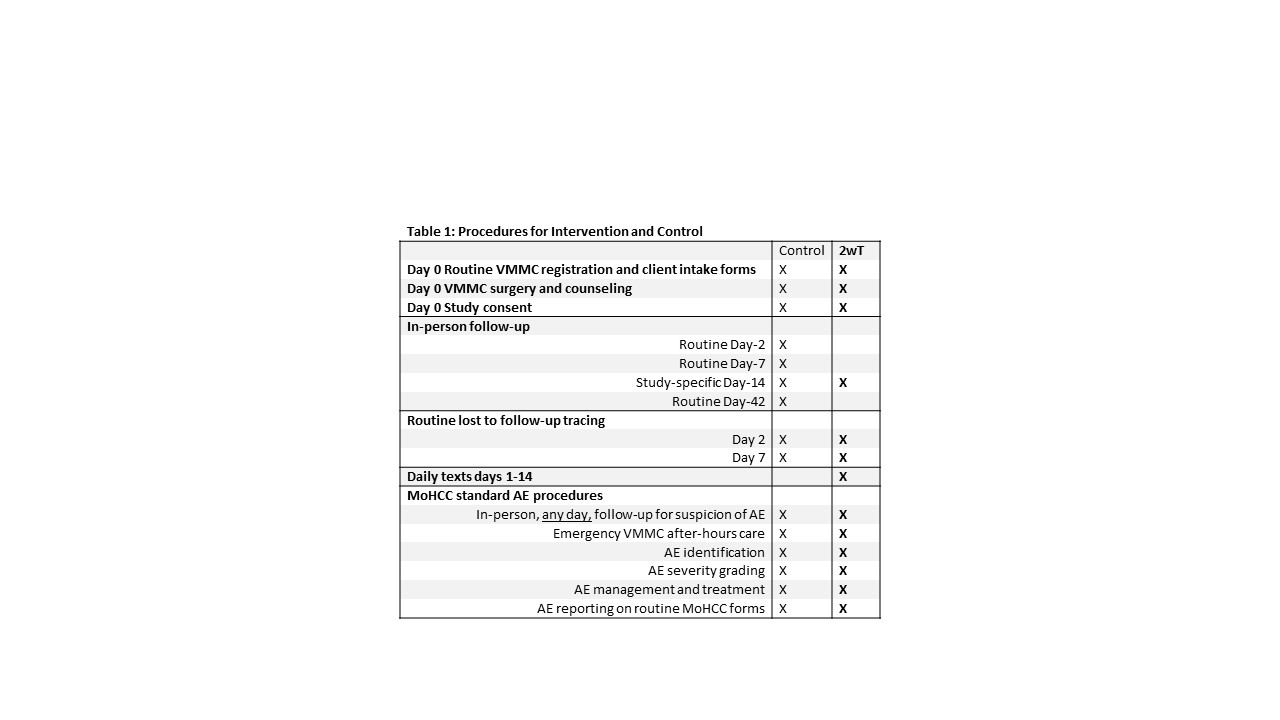
questionnaire administered by phone 42 days after circumcision. Men without cell phones and those who chose PrePex will be excluded as PrePex requires a device-removal visit 7 days after placement and has distinctly different follow-up protocols. As phone sharing practices are common, informed consent will ascertain whether eligible men have consistent access to a phone to receive messages about AEs. Participants who refuse enrollment will be asked to provide informed consent for data collection about reasons for non-participation. We expect to enroll and randomized 722 men into a 1:1 ratio of intervention and control within 6 months, allowing observation of the primary outcome by end of year 1. On Day 14, all men in both arms who return for follow-up will receive a $5 airtime credit.

Training of Study Personnel: VMMC staff at study sites will be informed of the study and briefed on study protocols. A 2wT study coordinator will be trained in confidentiality, protection of human subjects, enrollment, and data collection methods, working to not interfere with routine VMMC flow. For technology training, an experienced Medic Mobile trainer from Nairobi will work with the local study coordinator and team members in-person to implement and learn to maintain the Medic Mobile toolkit independently. We believe that achieving local maintenance and ownership of the system is feasible because of Medic Mobile’s considerable investments in open source collaboration and in designing software that is user friendly and intuitive enough to be adapted and maintained by local staff. As part of 2wT, one experienced VMMC nurse will serve as Study Coordinator in Harare and will manage the texting database, including text and voice follow-up communication with intervention clients. Zimbabwe-based co-investigators and interested partner organization researchers will also be invited for Medic Mobile training.

## Study Participants:

| Group name/description | Data collection method | Age range of subjects | Target number of individuals |
| --- | --- | --- | --- |
| Men in the intervention pilot (texting): A**ssigned texting** | Usability survey, routine VMMC clinical review | 18-65 | 50 |
| Men in intervention group (texting): **Randomized into group** | Interview, routine VMMC clinical review | 18-65 | 361(100 selected for the satisfaction and acceptability questionnaires) |
| Men in control group (routine care): **Randomized into group** | Routine VMMC clinical review | 18-65 | 361 |
| Healthcare workers (VMMC clinicians) | Interview | 18-65 | ~8 |

## Data collection:

**
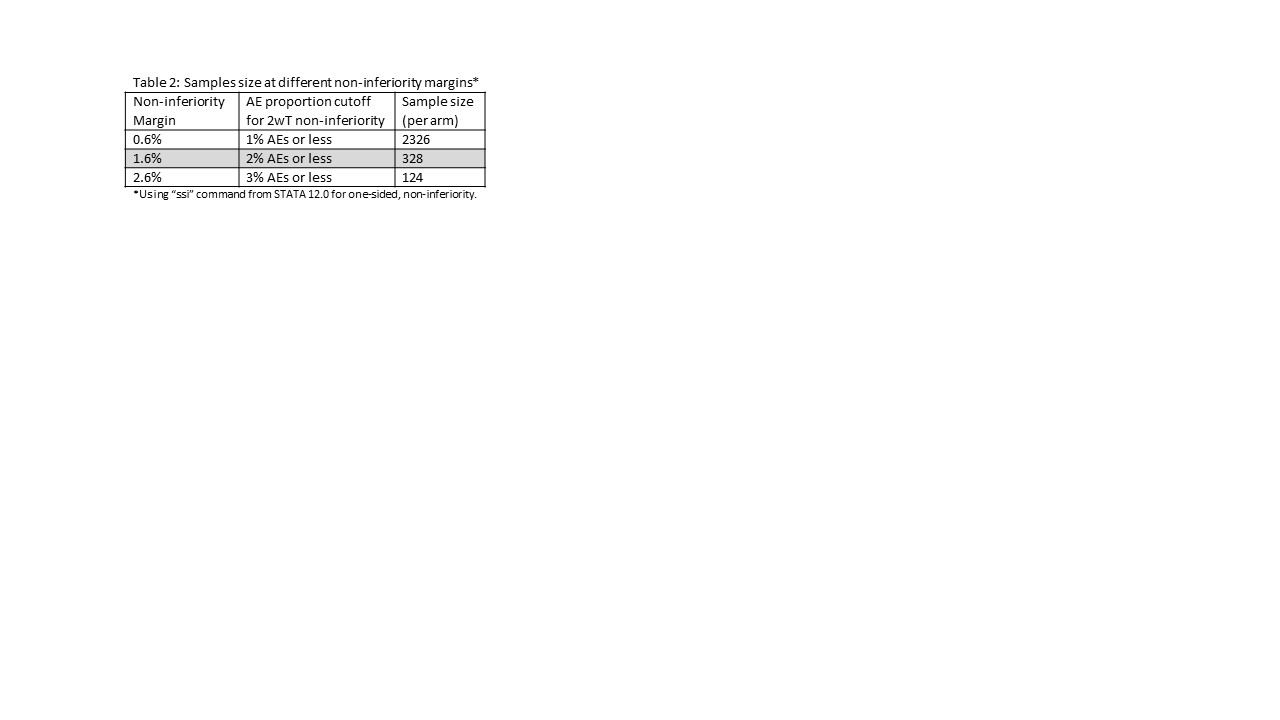
**Sample size: For Objective 1, the RCT will apply hypothesis testing for non-inferiority to examine the outcome of interest: AE rate (moderate or severe) occurring ≤ Day 14. A non-inferiority trial is appropriate as we wish to test our assumption that 2wT with reduced in-person follow-up is as safe (not clinically nor statistically inferior) as routine VMMC [59, 60], but with additional advantages including efficiency and lower direct and indirect costs to providers and patients. The non-inferiority margin, based on statistical and clinical considerations, is the maximum difference between the rate of AEs ≤14 Days in control and 2wT arms where we would conclude that 2wT is not inferior to routine care. We set that margin to 1.6% which would create a non-inferiority cutoff of 2% AEs or less in 2wT. The AE proportion cutoff for 2wT non-inferiority was set at a conservative 2% AEs to match the lower bound of AE rates reported by previous rigorous studies [1-3] and because an AE rate of 2% is regarded as a global standard of VMMC safety [61, 62], including in Zimbabwe [42]. Including a possible 10% LTFU, 361 participants per arm provides 90% power at an alpha of 2.5% to detect AEs greater than the non-inferiority margin of 1.6% (Table 2). We would conclude that 2wT is not inferior if we can rule out an AE rate of greater than 2%.

DATA ANALYSIS:

**For Objective 1**, the 2wT safety outcome of interest is cumulative AE rate (moderate or severe) ≤ Day 14. Incidence of AEs before Day 14 will be extracted from routine VMMC data for both 2wT and control. Incident AEs on Day 14 will be identified, classified, and graded for severity using routine MoHCC protocols [9] and recorded on routine VMMC AE forms. We will compare cumulative rates of any moderate or severe AE ≤ Day 14 between groups using Fisher’s exact test as the expected number of AEs is low. The rates will be calculated per arm as: (# moderate + severe AEs)/ (total # VMMC clients who attend/responded to 2, 7 or 14 Day follow-up visits or texts). Multivariate logistic regression models (any AE v none) will quantify the magnitude of difference, adjusting for any potential confounders. To determine follow-up visit reduction, we will compare the mean number of in-person visits for intervention and control using a t-test. A multivariate linear regression model will further quantify the effect of intervention on visit reduction, adjusting for potential confounders. Secondary outcomes include: AE rates on Day 14, texting response rate, time between 2wT AE text reporting and follow-up, severity of AEs. We will perform appropriate summaries of missing data and LTFU, testing for systematic differences in response by study arm. *We hypothesize that 1) 2wT is non-inferior to routine follow-up for patient safety and that 2) 2wT* *will reduce unnecessary follow-up over standard care.*

**For Objective 2,** 2wT costs, we will calculate the relative costs and outcomes (effects) of intervention versus control, including costs for technology, healthcare worker time, and client considerations (travel, text costs, missed work). We will conduct both activity-based costing from the implementation perspective and from the technology perspective to extrapolate our results as costs that would be incurred by the MoHCC should they elect widespread scale up of 2wT. **Approach for costing.** First we quantify the total direct and indirect costs of 2wT deployment, a method previously used for technology cost assessment in Malawi [47], which includes comprehensive costs from installation and training, routine maintenance, healthcare worker time associated with 2wT, and staff efficiency gains/losses in addition to the direct cost of technology. Second, we will estimate incremental costs (incremental relative to standard practice) for the intervention. This component entails a micro-costing study using activity-based approaches for costs incurred (trainings, VMMC service provision, follow-up) and costs averted (health costs for providers and patients saved by reducing visits), adapting previous VMMC costing estimates [63] for 2017 dollars when appropriate. Cost data will also be collected from the study budget, public health clinic budgets, published government reports, and the health economics literature. Lastly, to estimate the cost savings associated with 2wT over routine VMMC follow-up, we will conduct a time-motion study [64, 65] to quantify time spent for VMMC follow-up, indicating potential time savings for providers and VMMC clients. One trained observer will record VMMC client/provider follow-up interactions for 5 days, recording times and activities using a pre-established checklist and a personal digital assistant (PDA) that assigns start and end times. During these same 5 days, client time from registration through visit completion will be recorded by a second observer. Data will be exported into STATA 12.0 for analysis. The combined, overall costs for the delivery of 2wT in public health clinics will be estimated and compared to standard follow-up. *We hypothesize that* *2wT will reduce the costs associated with VMMC patient follow-up over standard care.*

**For Objective 3,** 2wT acceptability, using study recruitment and enrollment logs in addition to the texting database, we will describe levels of acceptance, participation, refusal and drop-out. We will carry out key informant interviews (KIIs) with up to 8 health care workers to gauge acceptability, satisfaction, identify facilitators and barriers to program success, and ascertain suggestions for intervention improvement. KIIs will be audio recorded and transcribed. Atlas.Ti software will be used to create a spreadsheet of key themes, perceived barriers, and suggested facilitators to the program from KIIs. We will also implement questionnaires at the Day 14 visit with a subset of 100 2wT VMMC clients in the main study who were randomized to texting to gauge satisfaction, estimate direct and indirect costs (time away from work, transportation costs), and ascertain suggestions for intervention improvement. Responses from these brief, self-administered, quantitative surveys with VMMC clients will be entered in Excel and frequencies explored in STATA 12.0. For feasibility, costing data will be combined with usability and acceptability information. These comprehensive data will be discussed at a final stakeholder meeting to disseminate study results, validate interpretations and refine recommendations for future scale up and modification of 2wT in Zimbabwe and beyond. Additional meetings between local researchers and the MoHCC will further determine feasibility for replication and scale-up. *We hypothesize that acceptability, usability, and feasibility will be high, aiding program scalability.*

## **Information management and analysis software**

During informed consent, participants will be asked to give permission to use data from their routine VMMC medical records, including the VMMC register and client intake form (CIF). Data from paper CIFs includes VMMC number, age, circumcision type, eligibility criteria, pre-procedure assessment, and adverse events during the procedure. Variables of interest from CIFs and register will also be entered using Medic Mobile software and verified through data checks, easing merging with the SMS database and smoothing data management. De-identified, coded data will be shared between researchers via secure networks.

Qualitative data will be entered, coded and analyzed as text documents in Atlas.ti 6. The first-line data quality assurance will be the responsibility of the I-TECH investigators. These files will also be sent to the team in Seattle, USA for secondary data quality assurance and analysis. The recordings of the interviews will be destroyed one year after the activity ends. However, the transcriptions will be kept for five years in compliance with University of Washington policy.

Capacity Building and Sustainability**:** ZAZIC is, with the exception of one in-county I-TECH employee, entirely run through true local organizations, demonstrating considerable experience in collaboration and capacity building. ZAZIC’s MoHCC partnership also enhances local ownership and buy-in, helping ensure replication of the mHealth intervention if successful. Frequent communication and team-based decision making among all members of the multinational, multidisciplinary team will help strengthen mHealth research capacity at I-TECH/Zimbabwe, University of Zimbabwe, and local NGO partner, ZiCHIRe. By working closely with researchers from the UW and Medic Mobile, Drs. Murenje and Tshimanga will gain mHealth research and implementation capacity, helping translate findings to public health policy. Through their leadership in this research, they will become mHealth leaders in Zimbabwe, creating a local team of researchers for scale-up and evaluation. Partnering with Medic Mobile in Nairobi, integrating with their existing software platform and building an expanded, local open source community will afford distinctive advantages for long-term maintenance and expansion. Dr. Murenje has already significantly contributed to research design and will continue to gain research skills from attendance at the mHealth conference in Year 2 to present results of and build research collaborations.

Future plans**:** If texting with VMMC clients is found to be non-inferior to routine, in-person follow-up for detection of AEs, scale up could dramatically reduce time spent on unnecessary VMMC follow-up visits by healthcare personnel and decrease the burden on VMMC clients. Future funding could enable scale-up of the intervention in other high volume VMMC sites with high mobile phone coverage, conducting additional rigorous evaluation in diverse field and clinical contexts. We would seek funding to test 2wT among rural populations or smaller urban areas. Lastly, we will explore how 2wT could be integrated into DHIS2, the national health information system, to improve and ease reporting.

Innovations

- Replace in-person follow-up visits with 2wT interaction between patients and providers holds potential to dramatically reduce the burden on the health system at low cost while maintaining patient safety. This rigorous research involving local researchers is needed to test and optimize the approach.
- Implement 2wT **within an existing MoHCC VMMC program structure** that will greatly improve the likelihood of program scalability and sustainability.
- Quantify expected MoHCC cost savings provided by 2wT scale-up
- Set a precedent by employing digital technology to identify cases of uncomplicated healing and thereby to reduce the burden of unnecessary VMMC clinic visits for both providers and patients - thus opening use of mHealth in post-operative care or other contexts with low rates of complications, potentially even long-term antiretroviral therapy use.
- As the rate of unnecessary, in-person care in VMMC programs is likely high, potential gains in efficiency identified through this low-cost digital innovation are large, increasing the likelihood of scale-up and replication in the region.

# 5. ETHICAL CONDUCT OF THE STUDY

This study will be conducted in compliance with the protocol after approval by the Medical Research Council of Zimbabwe (MRCZ) and the University of Washington IRB. No deviations from the protocol after approval will be implemented without prior approved amendment by these IRBs except where it may be necessary to eliminate any immediate hazard to study participants. In such case, the deviation will be reported to the IRBs as soon as possible.

**Risks:** For VMMC clients, male circumcision comes with some risk, but this study protocol does not cover the procedure which has its own consent. The VMMC procedure is not included in this study. As a routine medical preventive procedure, VMMC includes standard risks which are minimized and managed via MoHCC standard care and follow-up. All routine VMMC surgical procedures will be followed for this study.

Any additional MC-related risk that may be incurred by participants by virtue of their text-based follow-up will be monitored closely and managed as described above. Participants will be reminded that they may seek in-person follow-up if they suspect an AE or if they desire in-person follow-up without repercussion, confirming that participants understand that they may decline in-person care if they wish but are also welcome to seek it as per MoHCC guidelines. Referral cards for after hours and emergency care will be provided for 2wT participants as per routine care patients. Adverse event (AE) management will be conducted according to MoHCC guidelines, including assessment of the severity, type, and treatment plan. The Adverse Event guide is attached in an appendix. Participants will be allowed to seek in-person follow-up at any time, as with the control arm, as per MoHCC guidelines. Participants will also be informed that they can withdraw from the study at any time with no repercussions.

This study carries some small risk of social harms for VMMC clients. VMMC clients face a risk of stigma from circumcision as their circumcision status may be inadvertently revealed to others outside the study as a result of their participation or as a result of the texting intervention. VMMC clients who participate in surveys may feel uncomfortable answering questions about service quality and their opinions about the intervention.

Risks for HCW: This study carries minimal risk for HCW. Risks in this study are low relative to the potential for improvement in health and quality of healthcare services that could arise from the research. HCW will be informed that there are limited risk associated with the intervention and data collection procedures. Some HCW participating in interviews may feel uncomfortable answering questions about quality of health care services, HCW responsibilities, intervention quality, or other areas of inquiry.

Injury/side effects/adverse events: All routine VMMC surgical procedures will be followed for this study. Any additional MC-related risk that may be incurred by participants by virtue of their text-based follow-up will be monitored closely and managed as described above. Adverse event (AE) management will be conducted according to MoHCC guidelines, including assessment of the severity, type, and treatment plan. Participants will be allowed to seek in-person follow-up at any time, as with the control arm, as per MoHCC guidelines. Participants will also be informed that they can withdraw from the study at any time with no repercussions.

Discomfort/stress: Individuals who consent to participate may experience mild discomfort or stress as they will be asked to discuss topics that may be sensitive, including their VMMC method choice and rationale. We will prevent undue influence by stressing the voluntary nature of this study and each person’s ability to decline without any risk to their VMMC service quality.

Procedures for minimizing risk to participants include:

1. For AE management, AEs will be managed according to MoHCC guidelines. The 2wT study nurse will urge in-person follow-up for any 2wT intervention arm participant who is concerned about any possible AE. The participant will be instructed to return to the study site or the nearest hospital in case of any unexpected event, for example if there is severe discoloration of the penis, signs of infection (purulence), severe or increasing pain, wound opening, or any other significant concern that they may have. In the event of a complication meeting the case definition for a clinical AE associated with the VMMC, the provider will document the complication in the VMMC register and client intake form according to the guidelines. Mild post-operative infections will be managed with wound cleansing and dressing, and antibiotics will be prescribed where clinically indicated.
2. We will form an independent Data Safety and Monitoring Board (DSMB), see attached additional information. The DSMB will be available to review and advise on any concerning findings, such as social harms or other adverse events linked to the study, distress evoked among participants that might outweigh the potential benefits of the study, difficulties in recruitment, difficulties in study site participation, or any other issues that threaten the scientific or ethical foundation of the study. The DSMB will have the power to stop the study at any time. The investigators will also reach out to the UW IRB and members of the Medical Research Council of Zimbabwe in the event of concerns related to participant well-being or study validity, to consult on whether study procedures need to be modified or halted. There are likely no direct benefits to the participants for participation in this study. It is possible that the opinions expressed may inform future program changes.
3. Careful selection, training, and supervision of study personnel and site-level study liaisons, together with the systematic preparation of other HCW involved in study operations. Study personnel are selected for past training and ability to work with participants in this target population. All site-based study personnel (project site coordinator, data clerk) will be working specifically for this study from partner organization, ZICHIRe, and trained in procedures to ensure confidentiality in research settings. They will also be trained in research ethics and human subjects’ issues via the Collaborative Institutional Training Initiative (CITI) training. At each site, there will be a site-level study liaison (a VMMC provider) who is a regular staff member at the health facility who will be informed on study recruitment procedures, confidentiality protection, and documenting study operations. Dr. Feldacker will also take the Clinical Research Training On-Line Course for Principal Investigators and any additional courses suggested by University of Washington or NIH to further her understanding of ethical conduct of clinical research.
4. Study team members leading interviews and surveys are instructed to answer questions that may arise, and to refer all medical questions from patients to their health care providers. Study team members are skilled in dealing with anxiety or upset resulting from the collection of sensitive information. In the rare case that a participant becomes distressed during an interview or survey, the interviewer will reiterate the voluntary nature of participating in the research study and the rights of the participant to decline to respond to any question.

Benefits: There are limited or no direct benefits to participating in the study for HCW. However, if the intervention proves successful, future healthcare works could save invaluable time though the reduction of in-person VMMC review visits, freeing HCWs do perform VMMC or other duties. There are no direct benefits to the VMMC client. However, future clients could save time and money by only attending the reviews that were desired and forgoing compulsory reviews. This would ensure their safety and peace of mind while avoiding unnecessary follow-up. Information obtained in this study will be used by the MOHCC and its partners to inform follow-up visit schedules, potentially reducing in-person visits, which may lead to improving VMMC coverage and thus HIV prevention among uncircumcised men.

Invasion of privacy: HCW will be informed that there are limited physical risks and psychological risk associated with the intervention and data collection procedures. Some HCW participating in interviews may feel uncomfortable answering questions about quality of health care services, HCW responsibilities, intervention quality, or other areas of inquiry.

## TRAINING OF STUDY PERSONNEL

## VMMC staff at study sites will be informed of the study and briefed on study protocols. A 2wT study coordinator will be trained in confidentiality, protection of human subjects, enrollment, and data collection methods, working to not interfere with routine VMMC flow. For technology training, an experienced Medic Mobile trainer from Nairobi will work with the local study coordinator and team members in-person to implement and learn to maintain the Medic Mobile toolkit independently. We believe that achieving local maintenance and ownership of the system is feasible because of Medic Mobile’s considerable investments in open source collaboration and in designing software that is user friendly and intuitive enough to be adapted and maintained by local staff. As part of 2wT, one experienced VMMC nurse will serve as Study Coordinator in Harare and will manage the texting database, including text and voice follow-up communication with intervention clients. Zimbabwe-based co-investigators and interested partner organization researchers will also be invited for Medic Mobile training.

## Confidentiality:

Patients’ routine VMMC data from VMMC registers and client intake forms will be examined to extract data on patient demographics, clinical characteristics at baseline and during follow-up period, VMMC procedure notes, and adverse events reporting. This data will be entered into the study-specific database,

For the study specific database for analysis, only the study team will have access to participant identities for healthcare workers (HCW) and VMMC clients participating in interviews and surveys. Confidentiality will be maintained by assigning a unique study-specific identifying alphanumeric code to each participant’s data, for all interview and survey data collected as part of the study.

During the course of the study, study team members will review existing paper-based records at the site with identified patient data, including VMMC register and client intake forms as compared to data in the study data system for purposes of data quality assurance. Data will be abstracted only from the study database.

Any forms with identifying information, such as the informed consent forms and participant list with study-assigned identification numbers, will be kept separately from interview and survey data in locked cabinets or on password-protected, encrypted computers. Encryption will also be used wherever data are transferred. Implementation strategies for data management will take full consideration of regulations on confidentiality and security of electronic data transmission and storage. Upon completion of this study and exhaustion of the data retention period, all study identifiers will be deleted. In addition to the research staff’s maintenance of confidentiality, healthcare workers involved in the study will be oriented to the importance of confidentiality and their own roles in maintaining confidentiality.

The study database with study-specific data will include only coded information and no names or identifying numbers which could be used for individual identification of participants. Safeguarding confidentiality of personal data reported on study surveys and questionnaires will be achieved through identifier codes instead of full names on all research materials. The list referencing code number to name will be kept in a locked file cabinet in the research manager’s private office and coded data will be stored in computers in locked offices to which only project staff have access. The list relating names to number codes will be destroyed at the end of the study. Secondary data containing identifying information will be used to match records for patients who specifically consent to use of their health records, between the routine VMMC data and the study-specific database. Following the matching of data sing names and codes, the identifying information within the dataset abstracted from the secondary data sources will be destroyed and only the study-specific coded identifiers will be preserved. No individually identifiable information from VMMC clients or HCW will be published.

No individual identifiers will be recorded on notes or within the transcripts for HCW interviews. Only study personnel will have access to the consents, notes, and tapes. Tapes, when transcribed, will not have any identifying information other than a location number.

## CONSENT

Subject recruitment and consent will be in English and Shona. All staff are fluent in both English and local languages for translation and transcription of activity materials. Consent forms, advertisements, and study information materials will be translated by the local study team. Interviewer-administered forms will be used in English, as used in previous studies under the Zimbabwean protocol. Trained interviewers translate orally from English to Shona and complete forms in English. All study forms will be completed in English by study staff. Literacy in Zimbabwe is high and the study team is well versed is use of acceptable language level for VMMC clients.

Informed consent: The informed consent process is initiated prior to an individual agreeing to enroll in the cohort and continues throughout the individual’s participation in the cohort. Men who choose to enroll in the study will be provided with the enrollment informed consent, which describes in detail the study procedures and risks. Upon reviewing the document, the site staff member explains the research study to the subject and answers any questions that may arise. Extensive discussion of risks and possible benefits of study participation will be provided. The participant signs the informed consent document prior to the conduct of any study procedures. A copy of the enrollment consent form is given to the subjects for their records.

In obtaining informed consent, the study member enrolling patients will follow a strict script that emphasizes: 1) participation in the study is completely voluntary; 2) whether or not they participate will in no way adversely affect they care they receive. Consent procedures will occur before enrollment in the intervention study and before any interview or survey is administered. Participants in interviews or surveys will be informed of all study procedures, including audio recording and interview format/timings. Patients who seek VMMC services at the selected sites will not be consented unless they are being recruited to participate in the study. Participants in the study, whether HCW or VMMC client, may quit the study at any time without providing a reason. The amount of financial remuneration for the study participants (cell phone airtime equivalent of ~US$5) is regarded as compensation for time and travel and not considered an unfair enticement. HCWs will not be compensated.

Protection against Risk*:* Procedures for minimizing risk to participants include the careful selection, training, and supervision of study personnel and site-level study liaisons, together with the systematic preparation of other HCW involved in study operations. Study personnel are selected for past training and ability to work with participants in this target population. All site-based study personnel (project site coordinator, data clerk) will be working specifically for this study from partner organization, ZICHIRe, and trained in procedures to ensure confidentiality in research settings. They will also be trained in research ethics and human subjects’ issues via the Collaborative Institutional Training Initiative (CITI) training. At each site, there will be a site-level study liaison (a VMMC provider) who is a regular staff member at the health facility who will be informed on study recruitment procedures, confidentiality protection, and documenting study operations. Dr. Feldacker will also take the Clinical Research Training On-Line Course for Principal Investigators and any additional courses suggested by University of Washington or NIH to further her understanding of ethical conduct of clinical research.

Study team members leading interviews and surveys are instructed to answer questions that may arise, and to refer all medical questions from patients to their health care providers. Study team members are skilled in dealing with anxiety or upset resulting from the collection of sensitive information. In the rare case that a participant becomes distressed during an interview or survey, the interviewer will reiterate the voluntary nature of participating in the research study and the rights of the participant to decline to respond to any question.

The study database with study-specific data will include only coded information and no names or identifying numbers which could be used for individual identification of participants. Safeguarding confidentiality of personal data reported on study surveys and questionnaires will be achieved through identifier codes instead of full names on all research materials. The list referencing code number to name will be kept in a locked file cabinet in the research manager’s private office and coded data will be stored in computers in locked offices to which only project staff have access. The list relating names to number codes will be destroyed at the end of the study. Secondary data containing identifying information will be used to match records for patients who specifically consent to use of their health records, between the routine VMMC data and the study-specific database. Following the matching of data sing names and codes, the identifying information within the dataset abstracted from the secondary data sources will be destroyed and only the study-specific coded identifiers will be preserved. No individually identifiable information from VMMC clients or HCW will be published.

For AE management, AEs will be managed according to MoHCC guidelines. The 2wT study nurse will urge in-person follow-up for any 2wT intervention arm participant who is concerned about any possible AE. The participant will be instructed to return to the study site or the nearest hospital (in the case of dislodgement or severe pain) in case of any unexpected event, for example if there is severe discoloration of the penis, signs of infection (purulence), severe or increasing pain, wound opening, or any other significant concern that they may have. In the event of a complication meeting the case definition for a clinical AE associated with the VMMC, the provider will document the complication in the VMMC register according to the guidelines. Mild post-operative infections will be managed with wound cleansing and dressing, and antibiotics will be prescribed (see medications list) where clinically indicated.

## COMPENSATION FOR TIME:

No compensation will be provided for HCWs. $5 cell phone credit for both control and intervention arm on Day 14 visit.

LIMITATIONS AND UNCERTAINTIES:

Men undergoing PrePex VMMC are excluded, limiting generalizability to surgical VMMC. Phone ownership in Zimbabwe’s urban areas is high, but there is a small possibility that the population who undergoes VMMC has lower than estimated phone access, reducing the potential efficiency of a texting approach to follow-up. While evidence from Kenya and elsewhere suggests that patients are likely to interact with health professionals via SMS, it is possible that a higher than estimated percentage of clients will not respond to SMS, increasing the need for follow- up by voice calls, thus reducing the efficiency and cost gains afforded by the intervention. Lastly, we anticipate some challenges in program implementation such as network outages (though these are increasingly fewer and shorter) and user error, but previous Medic Mobile experience and the expert local team will enable swift identification of bottlenecks and appropriate, real-time, solutions.

## INTENDED USE OF FINDINGS

This project will evaluate the effectiveness, feasibility, and acceptability of a promising intervention to improve VMMC efficiency while maintaining patient safety. If successful, both providers and patients would neither perform nor attend unnecessary visits, providing distinct advantages to both groups. These advantages could be scaled up if the approach is adopted by the MoHCC, providing population-levels gains to millions of providers and patients of the VMMC program.

# Study Timeline

We expect the study to start immediately after the IRB approvals, technology adaptation, and pilot-driven modification. We aim to complete recruitment by December, 2017 in order to compete primary data analysis in March, 2018. Objectives 2 and 3, as well as much of the statistical analyses will be conducted in years 1 and 2.

# 7. Budget

This project will cost approximately $100,000. Budget attached.

# 8. References

1. Gray RH, Kigozi G, Serwadda D, Makumbi F, Watya S, Nalugoda F, et al. Male circumcision for HIV prevention in men in Rakai, Uganda: a randomised trial. The Lancet. 2007;369(9562):657-66.

2. Bailey RC, Moses S, Parker CB, Agot K, Maclean I, Krieger JN, et al. Male circumcision for HIV prevention in young men in Kisumu, Kenya: a randomised controlled trial. Lancet. 2007;369(9562):643-56. Epub 2007/02/27. doi: 10.1016/s0140-6736(07)60312-2. PubMed PMID: 17321310.

3. Auvert B, Taljaard D, Lagarde E, Sobngwi-Tambekou J, Sitta R, Puren A. Randomized, controlled intervention trial of male circumcision for reduction of HIV infection risk: the ANRS 1265 Trial. PLoS medicine. 2005;2(11):e298.

4. World Health Organization. WHO Progress Brief: Voluntary Medical Male Circumcision for HIV Prevention In 14 Priority Countries in East And Southern Africa2015 March 1, 2016. Available from: <http://apps.who.int/iris/bitstream/10665/179933/1/WHO_HIV_2015.21_eng.pdf?ua=1&ua=1>.

5. WHO U. Joint Strategic Action Framework to Accelerate the Scale-Up of Voluntary Medical Male Circumcision for HIV Prevention in Eastern and Southern Africa (2012–2016). Geneva: UNAIDS. 2011.

6. Njeuhmeli E, Forsythe S, Reed J, Opuni M, Bollinger L, Heard N, et al. Voluntary medical male circumcision: modeling the impact and cost of expanding male circumcision for HIV prevention in eastern and southern Africa. PLoS medicine. 2011;8(11):e1001132.

7. World Health Organization. Considerations for implementing models for optimizing the volume and efficiency of male circumcision services2010 [cited 2014 June 1]; Field testing edition: February 2010 Available from: <http://www.malecircumcision.org/programs/documents/mc_MOVE_2010_web.pdf>.

8. World Health Organization. Manual for male circumcision under local anaesthesia. Geneva: WHO. 2009.

9. President’s Emergency Plan for AIDS Relief. PEPFAR's Best Practices for Voluntary Medical Male Circumcision Site Operations: A service guide for site operations. 2013 May 2014. Available from: <http://www.malecircumcision.org/resource​s/documents/VMMC%20Best%20Practices03.04​.2013_web.pdf>. .

10. President's Emergency Plan for AIDS Relief. PEPFAR Monitoring, Evaluation, and Reporting Indicator Reference Guide. Washington DC: PEPFAR, 2015.

11. Campbell J, Dussault G, Buchan J, Pozo-Martin F, Guerra Arias M, Leone C, et al. A universal truth: no health without a workforce. Geneva: World Health Organization. 2013.

12. Phili R, Abdool-Karim Q, Ngesa O. Low adverse event rates following voluntary medical male circumcision in a high HIV disease burden public sector prevention programme in South Africa. Journal of the International AIDS Society. 2014;17(1).

13. Reed J, Grund J, Liu Y, Mwandi Z, Howard AA, McNairy ML, et al. Evaluation of loss-to-follow-up and post-operative adverse events in a voluntary medical male circumcision program in Nyanza Province, Kenya. Journal of acquired immune deficiency syndromes (1999). 2015.

14. Lebina L, Taruberekera N, Milovanovic M, Hatzold K, Mhazo M, Nhlapo C, et al. Piloting PrePex for Adult and Adolescent Male Circumcision in South Africa - Pain Is an Issue. PloS one. 2015;10(9):e0138755. Epub 2015/09/26. doi: 10.1371/journal.pone.0138755. PubMed PMID: 26405786; PubMed Central PMCID: PMCPMC4583405.

15. Brito MO, Lerebours L, Volquez C, Basora E, Khosla S, Lantigua F, et al. A Clinical Trial to Introduce Voluntary Medical Male Circumcision for HIV Prevention in Areas of High Prevalence in the Dominican Republic. PloS one. 2015;10(9):e0137376. Epub 2015/09/15. doi: 10.1371/journal.pone.0137376. PubMed PMID: 26367187; PubMed Central PMCID: PMCPMC4569265.

16. Kigozi G, Musoke R, Watya S, Kighoma N, Nkale J, Nakafeero M, et al. The safety and acceptance of the PrePex device for non-surgical adult male circumcision in Rakai, Uganda. A non-randomized observational study. PloS one. 2014;9(8):e100008. Epub 2014/08/22. doi: 10.1371/journal.pone.0100008. PubMed PMID: 25144194; PubMed Central PMCID: PMCPMC4140666.

17. Feldblum PJ, Odoyo-June E, Obiero W, Bailey RC, Combes S, Hart C, et al. Safety, effectiveness and acceptability of the PrePex device for adult male circumcision in Kenya. PloS one. 2014;9(5):e95357. Epub 2014/05/03. doi: 10.1371/journal.pone.0095357. PubMed PMID: 24788898; PubMed Central PMCID: PMCPMC4006910.

18. Ashengo TA, Grund J, Mhlanga M, Hlophe T, Mirira M, Bock N, et al. Feasibility and validity of telephone triage for adverse events during a voluntary medical male circumcision campaign in Swaziland. BMC Public Health. 2014;14:858. Epub 2014/08/20. doi: 10.1186/1471-2458-14-858. PubMed PMID: 25134856; PubMed Central PMCID: PMCPMC4150954.

19. Duffy K, Galukande M, Wooding N, Dea M, Coutinho A. Reach and cost-effectiveness of the PrePex device for safe male circumcision in Uganda. PloS one. 2013;8(5):e63134.

20. Herman-Roloff A, Bailey RC, Agot K. Factors associated with the safety of voluntary medical male circumcision in Nyanza province, Kenya. Bulletin of the World Health Organization. 2012;90(10):773-81. Epub 2012/10/31. doi: 10.2471/blt.12.106112. PubMed PMID: 23109745; PubMed Central PMCID: PMCPMC3471059.

21. Kohler PK, Namate D, Barnhart S, Chimbwandira F, Tippet-Barr BA, Perdue T, et al. Classification and rates of adverse events in a Malawi male circumcision program: impact of quality improvement training. BMC Health Serv Res. 2016;16(1):61. Epub 2016/02/19. doi: 10.1186/s12913-016-1305-x. PubMed PMID: 26888178; PubMed Central PMCID: PMCPMC4758015.

22. World Health Organization Regional Office for Africa. Progress in scaling up voluntary medical male circumcision or HIV prevention in Est and Southern Africa January-December 20122013 October 27, 2014. Available from: <http://www.malecircumcision.org/country_updates/documents/Progress%20in%20scaling%20up%20VMMC_Dec2013.pdf>.

23. Centers for Disease Control and Prevention. Voluntary medical male circumcision-southern and eastern Africa, 2010-2012. MMWR Morbidity and mortality weekly report [Internet]. 2013 August 19, 2016; 62(47):[953 p.]. Available from: <http://www.cdc.gov/mmwr/preview/mmwrhtml/mm6247a3.htm>.

24. Lankowski AJ, Siedner MJ, Bangsberg DR, Tsai AC. Impact of geographic and transportation-related barriers on HIV outcomes in sub-Saharan Africa: a systematic review. AIDS Behav. 2014;18(7):1199-223. Epub 2014/02/25. doi: 10.1007/s10461-014-0729-8. PubMed PMID: 24563115; PubMed Central PMCID: PMCPMC4047127.

25. Govindasamy D, Meghij J, Negussi EK, Baggaley RC, Ford N, Kranzer K. Interventions to improve or facilitate linkage to or retention in pre-ART (HIV) care and initiation of ART in low-and middle-income settings-a systematic review. Journal of the International AIDS Society. 2014;17(1).

26. Mukherjee JS, Ivers L, Leandre F, Farmer P, Behforouz H. Antiretroviral therapy in resource-poor settings: decreasing barriers to access and promoting adherence. JAIDS Journal of Acquired Immune Deficiency Syndromes. 2006;43:S123-S6.

27. Tuller DM, Bangsberg DR, Senkungu J, Ware NC, Emenyonu N, Weiser SD. Transportation costs impede sustained adherence and access to HAART in a clinic population in southwestern Uganda: a qualitative study. AIDS and Behavior. 2010;14(4):778-84.

28. Hardon AP, Akurut D, Comoro C, Ekezie C, Irunde HF, Gerrits T, et al. Hunger, waiting time and transport costs: time to confront challenges to ART adherence in Africa. AIDS care. 2007;19(5):658-65.

29. Odeny TA, Bailey RC, Bukusi EA, Simoni JM, Tapia KA, Yuhas K, et al. Text Messaging to Improve Attendance at Post-Operative Clinic Visits after Adult Male Circumcision for HIV Prevention: A Randomized Controlled Trial. PloS one. 2012;7(9):e43832. doi: 10.1371/journal.pone.0043832.

30. Cole-Lewis H, Kershaw T. Text Messaging as a Tool for Behavior Change in Disease Prevention and Management. Epidemiologic reviews. 2010;32(1):56-69. doi: 10.1093/epirev/mxq004. PubMed PMID: PMC3082846.

31. Health UDo, Services H. Using health text messages to improve consumer health knowledge, behaviors, and outcomes: an environmental scan. 2014. URL: <http://www> hrsa gov/healthit/txt4tots/environmentalscan pdf [accessed 2015-03-24][WebCite Cache]. 2015.

32. Déglise C, Suggs LS, Odermatt P. SMS for disease control in developing countries: a systematic review of mobile health applications. Journal of Telemedicine and Telecare. 2012;18(5):273-81.

33. Mbuagbaw L, Thabane L, Ongolo-Zogo P, Lester RT, Mills EJ, Smieja M, et al. The Cameroon Mobile Phone SMS (CAMPS) trial: a randomized trial of text messaging versus usual care for adherence to antiretroviral therapy. PloS one. 2012;7(12):e46909.

34. Pop-Eleches C, Thirumurthy H, Habyarimana JP, Zivin JG, Goldstein MP, De Walque D, et al. Mobile phone technologies improve adherence to antiretroviral treatment in a resource-limited setting: a randomized controlled trial of text message reminders. AIDS (London, England). 2011;25(6):825.

35. Kunutsor S, Walley J, Katabira E, Muchuro S, Balidawa H, Namagala E, et al. Using mobile phones to improve clinic attendance amongst an antiretroviral treatment cohort in rural Uganda: a cross-sectional and prospective study. AIDS and behavior. 2010;14(6):1347-52.

36. Odeny TA, Bailey RC, Bukusi EA, Simoni JM, Tapia KA, Yuhas K, et al. Effect of text messaging to deter early resumption of sexual activity after male circumcision for HIV prevention: a randomized controlled trial. Journal of acquired immune deficiency syndromes (1999). 2014;65(2):e50.

37. DeRenzi B, Findlater L, Payne J, Birnbaum B, Mangilima J, Parikh T, et al., editors. Improving community health worker performance through automated SMS. Proceedings of the fifth international conference on information and communication technologies and development; 2012: ACM.

38. Lester RT, Ritvo P, Mills EJ, Kariri A, Karanja S, Chung MH, et al. Effects of a mobile phone short message service on antiretroviral treatment adherence in Kenya (WelTel Kenya1): a randomised trial. The Lancet. 2010;376(9755):1838-45.

39. Perrier T, Dell N, DeRenzi B, Anderson R, Kinuthia J, Unger J, et al., editors. Engaging pregnant women in Kenya with a hybrid computer-human SMS communication system. Proceedings of the 33rd Annual ACM Conference on Human Factors in Computing Systems; 2015: ACM.

40. Holeman I, Evans J, Kane D, Grant L, Pagliari C, Weller D. Mobile health for cancer in low to middle income countries: priorities for research and development. European journal of cancer care. 2014;23(6):750-6.

41. Mahmud N, Rodriguez J, Nesbit J. A text message-based intervention to bridge the healthcare communication gap in the rural developing world. Technology and Health Care. 2010;18(2):137-44.

42. Zimbabwe Ministry of Health and Child Care. Accelerated Strategic and Operational Plan 2014 – 2018. Harare- Zimbabwe: 2014.

43. International Telecommunication Union. Mobile cellular subscriptions (per 100 people): The World Bank; 2015 [cited 2016 July 5]. Available from: <http://data.worldbank.org/indicator/IT.CEL.SETS.P2>.

44. ZAZIC. ZAZIC Monthly VMMC Programming Statistics. Harare: Unpublished; 2016.

45. President’s Emergency Plan for AIDS Relief. PEPFAR Monitoring, Evaluation, and Reporting Indicator Reference Guide. March 2015 ed. Washington DC: PEPFAR; 2015.

46. Marongwe P. Routine implementation of ZAZIC VMMC program. In: Feldacker C, editor. Key VMMC follow-up visit details from ZAZIC VMMC Program Officer ed. Harare2016.

47. Driessen J, Cioffi M, Alide N, Landis-Lewis Z, Gamadzi G, Gadabu OJ, et al. Modeling return on investment for an electronic medical record system in Lilongwe, Malawi. Journal of the American Medical Informatics Association. 2013;20(4):743-8.

48. Tomlinson M, Rotheram-Borus MJ, Swartz L, Tsai AC. Scaling up mHealth: where is the evidence? PLoS medicine. 2013;10(2):e1001382.

49. DeRenzi B, Borriello G, Jackson J, Kumar VS, Parikh TS, Virk P, et al. Mobile Phone Tools for Field‐Based Health care Workers in Low‐Income Countries. Mount Sinai Journal of Medicine: A Journal of Translational and Personalized Medicine. 2011;78(3):406-18.

50. Thirumurthy H, Lester RT. M-health for health behaviour change in resource-limited settings: applications to HIV care and beyond. Bulletin of the World Health Organization. 2012;90(5):390-2.

51. Banks K, McDonald SM, Scialom F. Mobile technology and the last mile:“Reluctant innovation” and FrontlineSMS. innovations. 2011;6(1):7-12.

52. Jumreornvong O. New Weapon In The War Against AIDS: Your Mobile Phone. Intersect: The Stanford Journal of Science, Technology and Society. 2014;7(1).

53. Medic Mobile. Medic Mobile For Antenatal Care: Do-It-Yourself Toolkit: Medic Mobile; 2016 [cited 2016 August 22]. Available from: <http://medicmobile.org/diy>.

54. West D. How mobile devices are transforming healthcare. Issues in technology innovation. 2012;18(1):1-11.

55. Pathak P. India Vaccination Pilot Progress Report. Medic Mobile and Clif Bar. 2012.

56. Medic Mobile. Drug Stock Monitoring: Medic Mobile; 2016 [cited 2016 August 22]. Available from: <http://medicmobile.org/usecases/drug-stock-monitoring>.

57. World Bank. Mobile cellular subscriptions (per 100 people), 1960-2014: The World Bank; 2016 [cited 2016 August 22]. Available from: <http://data.worldbank.org/indicator/IT.CEL.SETS.P2>.

58. Econet. Econet Wireless Tariffs (Incl VAT) Harare, Zimbabwe2016 [cited August 2016]. Available from: <https://www.econet.co.zw/services/tariffs>.

59. D'Agostino RB, Massaro JM, Sullivan LM. Non‐inferiority trials: design concepts and issues–the encounters of academic consultants in statistics. Statistics in medicine. 2003;22(2):169-86.

60. Schumi J, Wittes JT. Through the looking glass: understanding non-inferiority. Trials. 2011;12(1):1.

61. World Health Organization. WHO Technical Advisory Group on Innovations in Male Circumcision: Evaluation of two adult devices: Meeting report. Geneva, Switzerland: WHO, January, 2013 8/10/2015. Report No.

62. Byabagambi J, Kigonya A, Lawino A, Ssensamba JT, Twinomugisha A, Karamagi-Nkolo E, et al. A Guide to Improving the Quality of Safe Male Circumcision in Uganda2015 [cited 2016 August 12]. Available from: <https://www.usaidassist.org/sites/assist/files/uganda_guide_to_improving_the_quality_of_smc_a4_feb2015_ada.pdf>.

63. Schutte C, Tshimanga M, Mugurungi O, Come I, Necochea E, Mahomed M, et al. Comparative cost analysis of surgical and PrePex device male circumcision in Zimbabwe and Mozambique. Journal of acquired immune deficiency syndromes (1999). 2016;72(Suppl 1):S96.

64. Finkler SA, Knickman JR, Hendrickson G, Lipkin Jr M, Thompson WG. A comparison of work-sampling and time-and-motion techniques for studies in health services research. Health services research. 1993;28(5):577.

65. Were MC, Sutherland J, Bwana M, Ssali J, Emenyonu N, Tierney WM. Patterns of care in two HIV continuity clinics in Uganda, Africa: a time-motion study. AIDS care. 2008;20(6):677-82.

# 9. Appendices:

1. Study Budget
2. Consent form – men
3. Consent form – clinicians
4. SOP clinical team
5. SOP data management
6. SOP usability
7. DSMB charter
8. Recruiting flyer
9. Recruiting script
10. Clinician subject recruitment
11. Study procedures – visits
12. Texting flow chart
13. Brief survey participant
14. Follow up calls and texts scripts
15. Follow up calls/texts recording sheet
16. Qualitative interview clinicians
17. Study register for Day 14
18. Usability survey
19. MedicMobile Inbox example
20. Enrollment Form
21. Follow up Time and Motion Survey
22. Enrollment Register
