# Supplementary material for: Usability and acceptability of a two-way texting intervention for post-operative follow-up for voluntary medical male circumcision in Zimbabwe
Source: PLoS One. 2020 Jun 16;15(6):e0233234. doi: 10.1371/journal.pone.0233234 (PMC7297350; doi:10.1371/journal.pone.0233234)
Supplement: Attachment — Submitted filename: Manuscript Review PLOS - 16012020.docx [file pone.0233234.s003.docx]

**Manuscript Review – 16 January 2020**

Overall: Important topic and research results. The manuscript needs major editing to improve clarity.

**Abstract**

1. Objective that are not part of this paper are included. Need to focus only on what is included in the current manuscript. Other objectives of the overall study could be mentioned in the methods section.
2. Remove numbers on the aims – and they should only be two – according to the tittle: usability and acceptability of the 2wT
3. Conclusion: Findings on “men with concerns…” is not included in the results section of the abstract

**Main Manuscript**

*Introduction*

1. Lines 94 – 109 – should be part of the methods section
2. Lines 87 – 97 – should be in the setting section of the manuscript under methods
3. Lines 123- 124 – revise to indicate objective/aim of this study.

*Methods*

1. Theory of change section should be moved to the introduction section
2. Line 150 - 2wT Technology overview – reads like a descript of the intervention
3. Intervention – reads more like study procedures.
4. Line 176 – Only Shona and English were used in the testing, what about other languages like Ndebele. What was done if the participant did not understand any of those two languages (Shona and English)
5. Line 213 – Quantitative methods: Open-ended questions are part of qualitative research. Likert scale questions and demographics possible quantitative. It might be better in this section to put data collection from MC participants, and data collection from healthcare worker
6. Lines 231 – 236: Data analysis and the statistical analysis could be included in this section.

*Results*

1. Healthcare workers perspective – too long, and can be summarized highlighting the major findings

*Discussion*

1. Lines 410 – 415 – edited and be moved to study procedures or intervention description
2. Line 467 “it is clear that 2wT is warrants further” delete the word is
